# Optimizing network bandwidth slicing identification: NADAM-enhanced CNN and VAE data preprocessing for enhanced interpretability

**DOI:** 10.1371/journal.pone.0333286

**Published:** 2025-10-21

**Authors:** Md. Fahim Ul Islam, Shahriar Hossain, Md. Golam Rabiul Alam, Nafees Mansoor, Amitabha Chakrabarty

**Affiliations:** 1 Department of Computer Science and Engineering, BRAC University, Dhaka, Bangladesh; 2 Department of Computer Science and Engineering, University of Liberal Arts Bangladesh (ULAB), Dhaka, Bangladesh; Beijing Technology and Business University, CHINA

## Abstract

Communication networks of the future will rely heavily on network slicing (NS), a technology that enables the creation of distinct virtual networks within a shared physical infrastructure. This capability is critical for meeting the diverse quality of service (QoS) requirements of various applications, from ultra-reliable low-latency communications to massive IoT deployments. To achieve efficient network slicing, intelligent algorithms are essential for optimizing network resources and ensuring QoS. Artificial Intelligence (AI) models, particularly deep learning techniques, have emerged as powerful tools for automating and enhancing network slicing processes. These models are increasingly applied in next-generation mobile and wireless networks, including 5G, IoT infrastructure, and software-defined networking (SDN), to allocate resources and manage network slices dynamically. In this paper, we propose an Interpretable Network Bandwidth Slicing Identification (INBSI) system that leverages a modified Convolutional Neural Network (CNN) architecture with Nesterov-accelerated Adaptive Moment Estimation (NADAM) optimization. Additionally, we use a Variational Autoencoder (VAE) for preprocessing initial data, along with reconstructed data for data validity assessment. The model we propose outperforms other alternatives and reaches an accuracy peak of (84%) in the system environment. A range of accuracy was achieved by (k-nearest neighbors algorithm) KNN (76%), Random Forest (69%), BaggingClassifier (70%), and Gaussian Naive Bayes (GaussianNB) (55%). The accuracy of additional methods varies, including Decision Trees, AdaBoost, Deep Neural Forest (DNF), and Multilayer Perceptrons (MLPs). We utilize two eXplainable Artificial Intelligence (XAI) approaches, Shapley Additive Explanations (SHAP) and Local Interpretable Model-Agnostic Explanations (LIME), to provide insight into the impact of certain input characteristics on the network slicing process. Our work highlights the potential of AI-driven solutions in network slicing, offering insights for operators to optimize resource allocation and enhance future network management.

## 1 Introduction

In recent years, wireless communication systems have undergone remarkable advancements, driven by the ever-increasing demand for faster, more reliable, and efficient connectivity. The deployment of fifth-generation (5G) networks has already revolutionized the telecommunications landscape, enabling high-resolution data streaming (such as 4K and 8K), autonomous operations, telemedicine, smart cities, and immersive technologies like augmented reality (AR) and virtual reality (VR) [1,2]. However, as the world moves toward the fifth-generation (5G) and beyond era, the expectations for wireless communication systems have grown even further. 5G aims to push the boundaries of connectivity by reducing end-to-end latency, increasing data speeds, enhancing reliability, and creating a vast network of interconnected devices.

**Fig 1:**
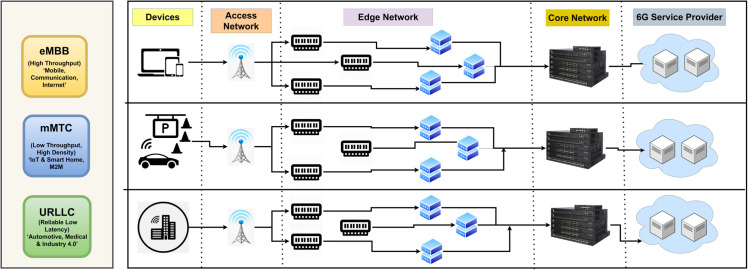
Network slicing architecture.

Despite the significant progress made by 5G, several challenges remain. For instance, while 5G networks achieve peak data speeds of up to 10 Gbps and latency as low as 1 ms, they still struggle to meet the diverse and smooth quality-of-service (QoS) requirements of emerging applications [3,4,52]. Moreover, traditional network architectures are often inadequate for handling the dynamic and complex demands of modern communication systems. This has led to the exploration of innovative solutions such as network slicing, which allows for the creation of virtualized, dedicated network resources tailored to specific applications or user groups. Fig 1 shows the general network slicing architecture. By leveraging technologies like software-defined networking (SDN) and network function virtualization (NFV), network slicing enables the efficient allocation of resources, improved scalability, and enhanced service customization [5–8]. Fig 1 refers to the comparison with other networks.

Therefore, network slicing is an essential technology enabling operators to generate various virtualized “slices” on a common physical network infrastructure [18]. Each slice is tailored to address particular application requirements, guaranteeing efficient resource utilization, scalability, and enhanced quality of service (QoS). For instance, the enhanced mobile broadband (eMBB) slice delivers high-speed data for bandwidth-heavy applications like AR and VR, while the massive machine-type communications (mMTC) slice connects billions of IoT devices in smart city scenarios such as environmental monitoring and smart homes. Similarly, the ultra-reliable low-latency communication (URLLC) slice provides ultra-low latency for critical applications like autonomous vehicles and remote surgery. Nevertheless, conventional 5G network slicing is predominantly static, featuring pre-established slices that cannot adjust in real time to fluctuating traffic requirements [19]. This rigidity may lead to performance complications, such as overburdening an eMBB slice during an abrupt AR/VR traffic surge or inadequately reallocating resources for latency-sensitive operations. As a result, static network slicing frequently results in inferior performance and user experiences, particularly in dynamic and essential situations.

To address these challenges, 5G introduces dynamic network slicing, enabling real-time adjustments to network slices based on evolving application needs [20]. Dynamic slicing persistently observes and reallocates resources in reaction to fluctuating traffic patterns, guaranteeing optimal network performance and consistent SLA adherence for various applications such as autonomous applications, high-bandwidth holographic communication, and ultra-dense IoT deployments. Therefore, network slice identification (NSI), which categorizes incoming traffic flows and assigns them to the appropriate slice based the application requirements [21]. For example, autonomous car data is sent to the URLLC slice for low-latency communication, IoT sensor data is sent to the mMTC slice for vast connectivity, and AR/VR applications are directed to the eMBB slice for high-bandwidth streaming.

However, the implementation of network slicing in 5G and beyond presents significant challenges, particularly in anomaly detection, data integrity, and resource optimization. Besides, effective decision-making relies on key parameters such as quality of service (QoS) and packet loss rate, which play a crucial role in maintaining network performance and efficiency. Existing solutions often fail to adequately address these issues, as they cannot detect anomalies in real time, ensure data integrity, and provide interpretable insights into network behavior. Furthermore, while machine learning (ML) techniques have been widely adopted to optimize network performance, their application in network slicing remains limited. The adoption of AI in network slicing faces three major challenges: inefficient resource allocation, lack of interpretability, and security vulnerabilities. Traditional ML models often struggle to dynamically allocate resources, leading to QoS violations in real-time network management. Additionally, these learning-based slicing models often function as black boxes, making it difficult to interpret how slices are assigned and adjusted. Furthermore, network slicing introduces security risks such as DDoS attacks and unauthorized access, which are challenging to detect in real time.

To address these challenges, our study proposes a novel approach that combines anomaly detection, data reconstruction, and interpretable AI models to optimize network slicing in 5G communications. Specifically, we introduce the use of variational autoencoders (VAEs) for anomaly detection and data reconstruction, enabling the identification of previously unknown network dynamics and ensuring data integrity. Additionally, we propose a lightweight convolutional neural network (CNN) model optimized with the NADAM algorithm, which is designed for efficient feature extraction, reduced overfitting, and optimal performance on resource-constrained devices. To improve transparency and ensure accurate decision-making, we further integrate explainable AI techniques, including SHAP (SHapley Additive exPlanations) and LIME (Local Interpretable Model-agnostic Explanations). These methods offer valuable insights into how our models make decisions, enhancing interpretability and trust in the slicing process.

The contributions of this paper are as follows:

- To the best of our knowledge, this is the first study to extensively utilize VAEs in network slicing configurations for anomaly detection and data reconstruction in 5G communications.
- We evaluate six classical ML models (KNN, Random Forest, Decision Tree, GaussianNB, BaggingClassifier, AdaBoost) and two DL models (Deep Neural Forest, MLP), and propose a lightweight CNN model with NADAM optimization for superior performance and efficiency in resource-constrained environments.
- We introduce SHAP and LIME to interpret the results of the XGBClassifier and MLP models, enhancing the transparency and practical applicability of our approach in 5G and 6G scenarios.
- We assess the efficiency of our models using metrics such as Mean Squared Error (MSE), Mean Absolute Error (MAE), accuracy curves, loss curves, confusion matrices, and classification reports, demonstrating the superiority of our proposed framework in network slicing scenarios.

The remainder of this paper is organized as follows. Sect 2 provides an overview of network slicing and related works in this domain. Sect 3 presents our proposed approach for effective network slicing. Sect 4 describes the implementation and evaluation techniques used in this study. Sect 5 analyzes the results and insights obtained from our experiments. Sect 6 develops a use case for our model in different applications. Finally, Sect 7 concludes the paper and discusses future directions.

By addressing the limitations of existing solutions and introducing innovative techniques for anomaly detection, data reconstruction, and interpretable AI, this research aims to enhance the quality of service (QoS) and user experience (QoE) in 5G and 6G networks, paving the way for more efficient and reliable wireless communication systems. Table 1 shows a comparison between 5G and other networks.

**Table 1:** Comparison of 5G and other networks.

Aspect	5G	6G
Peak data rate	10 Gbps	1 Tbps
Frequency	3–300 GHz	1000 GHz
Latency	10 ms	less than 1 ms
Mobility support	Up to 500 km/h	Up to 1000 km/h
Spectral efficiency	30-40 bps/Hz	100 bps/Hz

## 2 Literature review

In the realm of network slicing, where SDN and NFV play a pivotal role, extensive research efforts have been undertaken to achieve optimal slicing solutions that cater to the diverse needs of various applications and services within 5*G* and 6*G* networks. In this section, we present a structured overview of the available literature, with a primary emphasis on research contributions relevant to the implementation of network slicing through the utilization of SDN and NFV technologies. It is important to emphasize that ML and DL approaches represent a relatively novel and innovative approach within this sector, introducing a fresh viewpoint to the discipline.

Robert et al. [9] offered a novel approach for low-latency network slicing. They adapted the A* method to add QoS parameters and outperformed Dijkstra’s algorithm with a precalculated heuristic function and real-time congestion management. The approach was included in an SDN module termed a route computation element for optimizing network slice pathways. It’s vital to note that this paper did not build a data anomaly detection or an automated computational classifier/predictor algorithm. However, it is important to investigate the problem for efficient slice distribution.

Numerous research works have addressed the theoretical topic offered here. While network slicing is commonly conducted in an orthogonal multiple access (OMA) fashion, Yuanwen et al. [10] dives into the possibilities of rate-splitting multiple access (RSMA), enabling improved flexibility and superior data rate capabilities. RSMA can outperform both OMA and non-orthogonal multiple access (NOMA), rendering it a viable solution for network slicing across varied regions. Besides, Asmaa et al. [11] analyzes the integration of network slicing into cooperative NOMA-based systems with underlay device-to-device (D2D) connections. The research establishes an optimization issue that seeks to increase system throughput while meeting slice requirements. By adopting a two-stage resource allocation technique based on the swapping-based matching hypothesis, what is suggested regularly surpasses others when it comes to both system throughput and the number of enabled D2D pairs. However, it is crucial to highlight that this study, although offering simulated data, does not offer a real-time execution of its proposed techniques. Consequently, it is essential to address the actual execution of this work at a production level, particularly on edge devices, while assuring the preservation of data privacy.

This study by Gharehgoli et al. [12] highlights network slicing in the context of 5G and beyond, addressing information ambiguity, including demand issues. It employs deep reinforcement learning (DRL) for optimizing resource allocation for communication services. Additionally, Gupta et al. [13] investigated network slicing in a radio access network with industrial Internet of things (IIoT) devices sharing infrastructure. It utilized deep reinforcement learning for resource orchestration to meet varying service demands. The study concentrated on reasoning decision objectives, such as maximizing system efficiency, spectral effectiveness, agreements on service levels, packet rate, and minimizing power consumption and transmission latency. It leveraged generative adversarial network-based deep distributional noisy Q-networks (GAN–NoisyNet) and introduced dueling GAN–NoisyNet to improve the making of decisions . However, it’s worth taking into account that the study does not highlight interpretable network slice allocation or explanations of AI concepts, which are also significant concerns.

Furthermore, to make the slice more distributed, automated systems have been proposed in many studies. To boost service quality and optimize network slicing, Dangi et al. [14] integrated artificial intelligence and ML approaches. The methodology involved three essential phases: dataset loading, hyperparameter optimization using harmony search optimization (HHO), and network slice classification with a hybrid DL model, combining HHO, CNN, and long short-term memory (LSTM). This approach sought to increase QoS and network slicing efficiency. Furthermore, the study attempted to bridge the gap in NS architectures by presenting the slicing future internet infrastructures (SFI2) architecture. This unique strategy emphasizes the integration of experimental networks and incorporates ML for native optimizations, energy-efficient slicing, and security suited to NS. Joberto et al. [15] utilized ML approaches to optimize all phases of network slicing, from orchestrating to resource prediction. Particularly, it focused on resource allocation strategies, increasing sustainability, and reducing energy consumption during various phases of the network slice lifespan. However, it is imperative to highlight that the model lacks a priority approach in identifying the network functions that contribute most efficiently to slice distribution and in generating reconstructed data to reduce data mistakes.

Focusing on load balancing and slice failure management, many approaches have been proposed. Najwan et al. [16] introduced an intelligent recurrent neural network (RNN) controller as well as the intelligent SDN multi-spike neural system (IMSNS) for service type identification, leveraging moderately multi-spike return neural networks (MMSRNN) and time-based coding to reduce energy consumption and improve traffic identification for optimized network slice allocation. Additionally, Michael [17] proposes a data-driven ML-driven slicing and allocation model that mainly enabled improved quality of service and traffic-aware trustworthy dynamic slicing, where tools can be strategically allocated and redirected between network slices depending on time-dependent virtual resource demands in the paper. Additionally, the study utilized supervised ML techniques to develop an SLA deconstruction generator for network slicing, evaluating the accuracy, sample complexity, and model explainability of each algorithm class. Following with approach, Anurag et al. [22] proposed Adaptive Learning as the ‘ADAPTIVE6G’ framework as a unique approach to network slicing design, concentrating on resource management and load prediction in data-driven Beyond 5*G* wireless networks. The system leverages knowledge collected via transfer learning (TL) methodologies and is evaluated for solving difficult network load estimate challenges, with the ultimate goal of establishing a more equitable and balanced distribution of network resources. Also in the context of reconfigurable wireless network solutions,Sulaiman et al. [23] introduced a hybrid DL model that combined CNN for tasks like resource allocation, network reconfiguration, and slice selection, and LSTM for handling statistical information related to network slices, including load balancing and error rates. Nevertheless, all these research studies fall short in terms of providing transparency in network slice allocation and outlining the underlying distributed secure data. These elements hold important importance in the context of optimizing prediction algorithms for the study’s objectives.

Sarder et al. [24] proposed a fog load balancing framework utilizing NB-IoT and game theory for massive Machine Type Communication (mMTC) applications. They articulated the issue as a bankruptcy game, utilizing Shapley value-based scheduling with a low-complexity Greedy Iterative Time Scheduling (GITS) algorithm. The fog load balancing was formulated as a Hitchcock–Koopmans transportation problem and resolved with Vogel’s Approximation Method (VAM), substantially decreasing job balancing expenses. Madyan et al. [25] proposed a risk-sensitive resource allocation strategy for ultra-reliable low-latency communication traffic in 5G New Radio networks. They optimized resource allocation utilizing Conditional Value at Risk (CVaR) and Markov’s inequality, so ensuring the reliability of Ultra-Reliable Low Latency Communication (URLLC). The issue was subdivided into subproblems and resolved iteratively, resulting in effective URLLC scheduling while preserving eMBB reliability.

Anupam et al. [26] examined eMBB-URLLC co-scheduling employing the puncturing technique to optimize latency, reliability, and spectrum efficiency. They formulated an optimization problem maximizing the Minimum Expected Achieved Rate (MEAR) of eMBB users, applying a Penalty Successive Upper Bound Minimization (PSUM) algorithm for eMBB and an Optimal Transportation Model (TM) for URLLC scheduling. Their methodology surpassed baseline methods in MEAR and fairness. Madyan et al. [27] evaluated dynamic resource slicing for eMBB and URLLC services, seeking to maximize eMBB data rates while assuring URLLC reliability. They provided a framework for Deep Reinforcement Learning (DRL) enhanced by optimization, segmenting the problem into subproblems, and converting them into convex forms. The technique successfully equilibrated URLLC limitations while preserving eMBB reliability over 90%.

Table 2 has been generated to offer a summary of the methods that have been applied for network slicing and the classification of expected services. Upon a detailed evaluation of existing works, it becomes obvious that these efforts generally revolve around boosting system efficiency. Yet, there are considerable gaps in addressing the essential topic of guaranteeing data safety during real-time analysis, with a focus on minimizing the introduction of new errors. Additionally, maintaining low data errors in data produced from the source is necessary for sustaining data credibility while preserving the underlying data correlations. Moreover, it is worth mentioning that certain network characteristics play a vital role in the classification and distribution of slices, making them indispensable for establishing system awareness and enabling improved decision-making without the need for human interaction. Our contributions generally center on addressing these key views.

**Table 2:** Overview of the existing related models.

Research	Dataset	Algorithms	Key Insights
Robert et al. [9]	N/A	Proposed Arch.	Modified A* improves 5G latency & time
Yuanwen et al. [10]	N/A	RSMA	Flexible decoding & higher rates
Kostos et al. [11]	N/A	NOMA	Coop. NOMA boosts slicing throughput
Amir et al. [12]	N/A	RDPG	Handles CSI uncertainty effectively
Amir et al. [13]	N/A	GAN–NoisyNet	Enhances throughput & energy in IIoT
Ramraj et al. [14]	Unicauca v2, 5G slice	KNN, CNN, LSTM, HHO, SVM	HHO-CNN+LSTM outperforms classic ML
Joberto et al. [15]	DDoS-2019	KNN, RF, SVM, MLP	ML slicing improves energy & security
Najwan et al. [16]	NS Dataset (65K)	MMSRNN, CNN	MMSRNN aids traffic & energy efficiency
Michael et al. [17]	Domain Emulator	RF, GB, CNN	GB + NN work well in slicing
Anurag A et al. [22]	NS Dataset (65K)	ADAPTIVE6G	TL-based ADAPTIVE6G improves load est.
Sulaiman et al. [23]	NS Dataset (65K)	CNN+LSTM	Achieves 95.17% accuracy for 5G/6G
**Ours**	NS Dataset (65K)	CNN, LIME, SHAP, VAE	VAE detects anomalies, CNN classifies, SHAP/LIME for explainability

## 3 System framework and use case scenario

**Fig 2:**
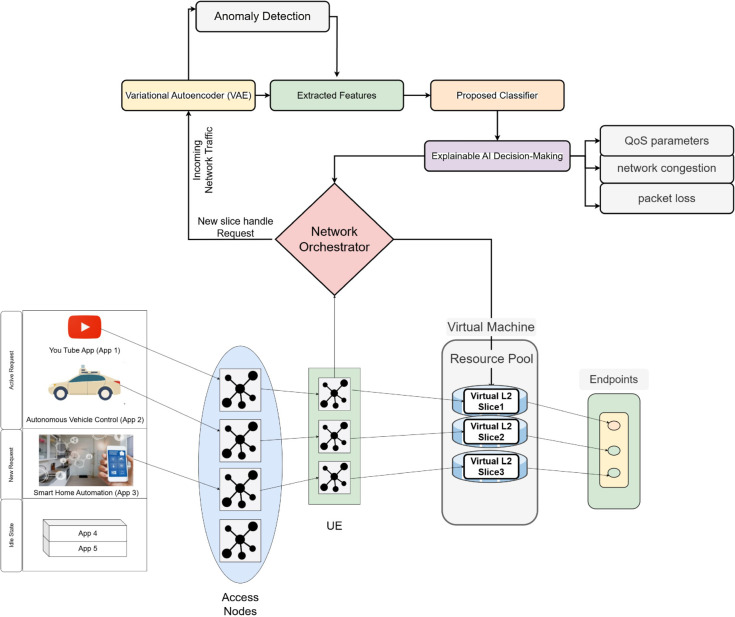
Our INBSI model for 5G network slicing, utilizing VAE for anomaly detection, CNN with NADAM for slice prediction, and SHAP/LIME for interpretability to ensure efficient resource allocation and QoS compliance.

Our INBSI model provides a holistic solution for optimal resource allocation in 5G network slicing by dynamically regulating network resources according to application-specific demands shown in Fig 2. Upon the initialization of a 5G network, active applications such as App 1 (e.g., video streaming) and App 2 (e.g., VoIP services) are allocated to designated network slices based on their Service Level Agreement (SLA) criteria, including bandwidth, latency, and quality of service (QoS). A default slice is reserved for unclassified or unknown traffic, ensuring no traffic is left unmanaged.

Upon the connection of a new application, such as App 3 (a real-time gaming application), to the network via User Equipment (UE) and Access Node, the Network Orchestrator first allocates it to the default slice. This assignment is temporary. The traffic from App 3 is initially processed by a VAE model, which identifies abnormalities in the flow and reconstructs any distorted or missing data. This procedure guarantees that only trustworthy, clean data is utilized for further research. Once the data is cleaned, the system extracts critical network metrics such as bandwidth utilization, latency, and packet loss using our suggested CNN model, which is optimized with the NADAM optimizer. This model is explicitly engineered to manage the complexities of network traffic by capturing temporal and spatial dependencies within the data. These attributes are utilized to ascertain if App 3 can persist in the default slice or whether a new specialized slice is required. If our suggested CNN model finds that App 3 requires a fresh slice, the system employs SHAP and LIME to make the choice clear. These XAI approaches assist explain why a fresh slice is needed by emphasizing crucial issues such as the application’s high QoS needs, network congestion, and packet loss that exceed acceptable norms. This interpretability aids stakeholders in comprehending the rationale for dynamic slice allocation, hence fostering trust in the system’s judgments.

**Fig 3:**
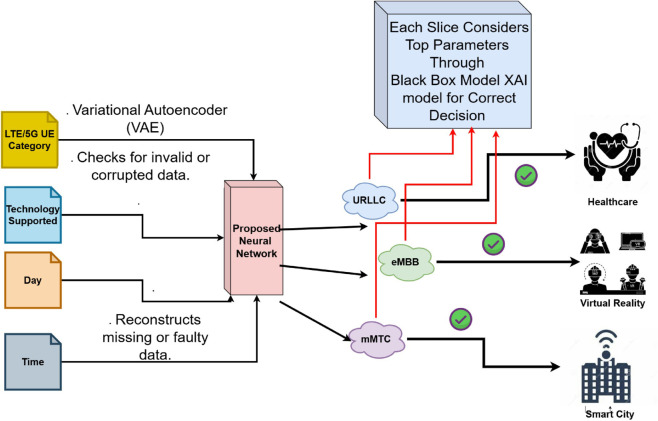
Graphical representation of our proposed INBSI system.

According to the prediction, the Network Orchestrator dynamically establishes a new dedicated slice (Slice 3) utilizing resources from the virtualized Resource Pool. The orchestrator reallocates the required resources, such as bandwidth and computing power, to ensure that Slice 3 is optimized for the requirements of App 3. The application’s traffic is subsequently transferred from the default slice to Slice 3, guaranteeing maximum performance for real-time gaming. Once App 3 disconnects or becomes inactive, Slice 3 is terminated, and its resources are returned to the Resource Pool, ensuring that network resources are utilized efficiently for future applications. Throughout this process, the system continually analyzes the performance of Slice 3 to ensure that it matches the application’s increasing demands. If the needs of App 3 vary such as during peak gaming hours, the Network Orchestrator may alter the slice’s resources to maintain optimal performance. This dynamic resource adjustment guarantees that the network can react to fluctuating traffic circumstances and application demands in real-time. The overall working mechanism of the INBSI system is illustrated in Fig 3. It begins by applying the Variational Autoencoder (VAE) for data anomaly detection and preprocessing. The processed data is then passed to the proposed Convolutional Neural Network (CNN) model, which classifies the network slicing accurately. Finally, based on the model’s parameters, decision-making is enhanced through the application of Explainable AI (XAI) methods.

## 4 Proposed Interpretable Network Bandwidth Slicing Identification (INBSI) system

**Fig 4:**
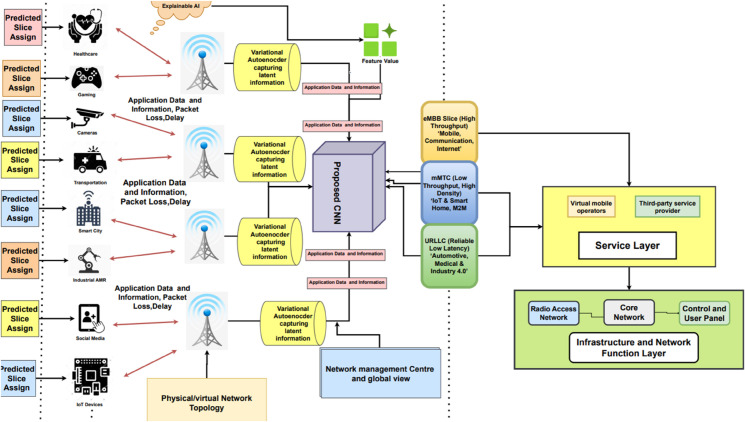
The proposed Network Bandwidth Slicing Identification (INBSI) system’s top-level paradigm.

Our proposed Interpretable Network Bandwidth Slicing Identification (INBSI) system efficiently mitigates data abnormalities within network infrastructure by integrating feature importance analysis with precise slice assignment. Operating primarily in the application layer (Fig 4), our system leverages a VAE model to detect errors and encode registration-related data, while a custom CNN model classifies and predicts scattered slices based on user input, packet loss, QoS Class Identifier (QCI), and other parameters. Through the use of explainable AI (XAI) techniques, network operators can better understand and assess the fairness, accuracy, and performance of slice allocation decisions. XAI improves interpretability by assigning accurate priorities to specific slices using feature importance, enhancing load balancing and dynamic traffic management.

**Fig 5:**
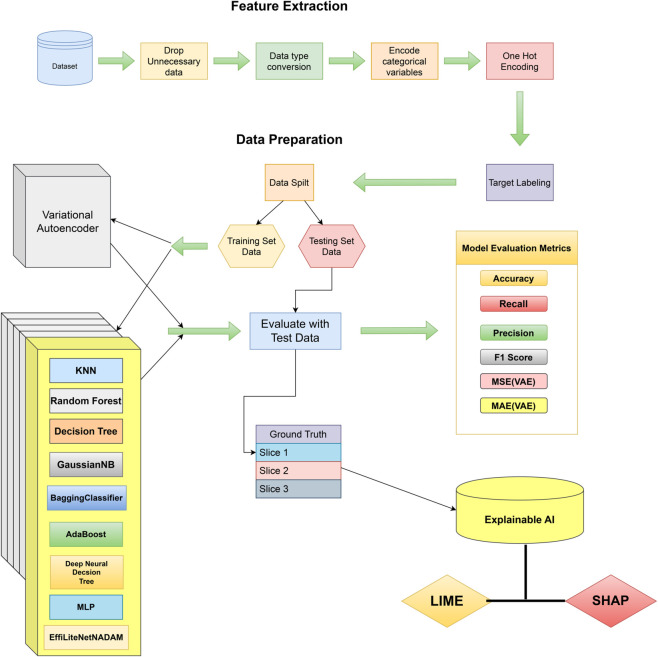
Working methodology of our proposed INBSI system.

We evaluate how our models behave in our proposed INBSI system environment using learning curves, classification reports, confusion matrices, training accuracy, and loss curves. For a thorough evaluation of model outcomes, we also calculate mean squared error (MSE) and mean absolute error (MAE). The proficient slice distribution is illustrated in Fig 5.

### 4.1 Dataset

This section describes the dataset and the preprocessing steps that the dataset went through before being fed to the proposed model.

#### 4.1.1 Data collection.

The dataset was collected from Kaggle, “DeepSlice and Secure5G - 5G and LTE Wireless Dataset” [28]. The 6*G* data is still not available in this domain, so we used the 5*G* dataset for performance measurement. The dataset comprises the most important network and device KPIs, such as the kind of connected device (such as a smartphone, IoT device, URLLC device, etc.), the User Equipment (UE) category, the QoS class identifier (QCI), the packet delay budget, the maximum packet loss, the time and day of the week, etc. Control packets between the UE and network, which mainly contain more than 65,000 different input combinations with target labels can be used to collect these KPIs [29],[30].

#### 4.1.2 Training and testing data.

To specify the model, we begin by splitting the dataset using a VAE, establishing distinct training and test sets in a 70:30 ratio. Subsequently, we train a suite of varied machine learning classifiers, including KNN, Random Forest, Decision Tree, GaussianNB, Bagging classifier, AdaBoost, DNF, MLP, and our innovative proposed CNN model, on the meticulously generated dataset. Our pick of the most effective model is based on its ability to predict network slicing labels. Finally, we enrich the dataset with explainable AI by combining classifier models.

### 4.2 Preprocessing steps

The dataset underwent a series of preprocessing steps to ensure its suitability for the specified machine learning models, beginning with the removal of unrelated columns that did not contribute to the predictive performance. The preprocessing began with the removal of unrelated columns that did not contribute to the predictive performance. To maintain uniformity in data representation, integer values within categorical columns, specifically “LTE/5G UE Category (Input 2)” and “QCI (Input 6)”, were transformed into string format. This step was crucial for preventing potential discrepancies in data type interpretation during model training. Subsequently, categorical variables were encoded using a label encoding technique to convert non-numeric features into numerical representations, thereby enhancing the model’s ability to process and learn from these attributes. The features subjected to label encoding included “Use Case Type (Input 1)”, “LTE/5G UE Category (Input 2)”, “Technology Supported (Input 3)”, “Day (Input 4)”, and “Slice Type (Output)”, ensuring a standardized numerical representation of categorical data. Furthermore, to optimize model performance and reduce potential noise, two distinct features were eliminated from the dataset based on their irrelevance or low contribution to predictive accuracy. These preprocessing steps - including the ethical handling of imbalanced medical data through [specific techniques] - collectively enhanced data consistency, privacy preservation, and compatibility, ultimately contributing to the robustness and efficiency of the learning model while maintaining compliance with healthcare data protection regulations.

### 4.3 VAE for continuous latent space representation

**Fig 6:**
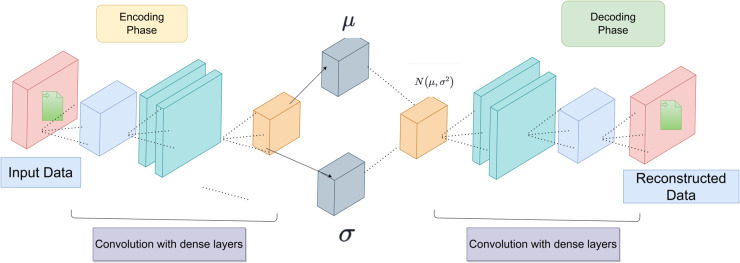
Variational autoencoder architecture.

In the context of data anomaly detection and error pattern identification, we propose the use of a VAE model. By duplicating input data, the VAE technique is applied to the autoencoder that learns a continuous latent space representation. In addition to an encoder and a decoder network, reconstruction likelihood is also used as a probabilistic measure [31]. Through learning mappings from low-dimensional latent vectors to high-dimensional inputs, VAEs approximate this manifold while encouraging global structure in the latent space [36]. The architecture of VAE is shown in Fig 6. The VAE employs a dense-layer encoder architecture, batch normalization, dropout regularization, and a unique loss function to optimize latent space dimensions and reduce input data dimensionality.

#### 4.3.1 Gaussian encoding.

The encoder outputs the set of parameters of a distribution in the latent space given an input data point *x*. To make computations easier, it is usual to assume that this distribution is Gaussian and has a diagonally drawn covariance matrix. The encoder predicts the mean and log-variance of this Gaussian distribution as *μ* and $\sigma^{2}$, accordingly. The encoder can be expressed as follows if *z* is the latent variable (sampled from the latent space distribution):

$$h=\tanh(w_{h}+b_{h}),$$ (1)

$$\mu =w_{\mu }h+b_{\mu },$$ (2)

$$\sigma^{2}=\exp(w_{\sigma^{2}}h+b_{\sigma^{2}}),$$ (3)

$$q(z|x)=N\left(z;\mu ,\sigma^{2}I\right).$$ (4)

#### 4.3.2 Reparameterization.

We take a sample from the distribution with *μ* and $\sigma^{2}$ as parameters to produce the latent variable *z*. After sampling from a typical Gaussian distribution with mean 0 and variance 1, the reparameterization approach is used to modify the data points.

$$z=\mu +\epsilon \cdot \exp\left(\frac{1}{2}\log(\sigma^{2})\right),$$ (5)

#### 4.3.3 Decoding.

The decoder takes the sampled latent variable *z* and attempts to reconstruct the original data point *x*. The decoder is typically a neural network that outputs the reconstruction.

#### 4.3.4 Variational inference.

The primary goal in variational inference is to minimize the Kullback-Leibler (KL) divergence between the approximating distribution q(z∣x), with parameters *ϕ*, and the true posterior distribution p(z∣x), with parameters *θ*, as presented in [37].

$$\phi^{*},\theta^{*}=\arg\min_{\phi ,\theta }KL(q(z|x^{\left(i\right)};\phi )\|p(z|x^{\left(i\right)};\theta )),$$ (6)

The KL divergence between two probability distributions *q*(*z*) and *p*(*z*) is defined as:

$$KL(q\|p)=\int q(z)\log\frac{q(z)}{p(z)}\;dz,$$ (7)

where it estimates the difference in information richness between two distributions.

The KL divergence is described in terms of expectancies as,

KL(q‖p)=E[logq]−E[logp]+logp, (8)

while demonstrating how it may be calculated as the difference between the expected value of the logarithm of *q*(*z*) and the expected value of the logarithm of *p*(*z*), plus *log*(*p*).

KL(q‖p)=ELBO+logp, (9)

The evidence lower bound (ELBO) is introduced as an important concept. It represents a lower bound on the log-likelihood of the data. The relationship between the KL divergence, ELBO, and the log-likelihood term is shown as follows.

ELBO=−KL(q‖p)+E[logp], (10)

The ELBO is decomposed into two parts by this equation: the negative KL divergence term and the predicted value of the logarithm of *p*(*z*). Finally, we combine the goals of determining the best parameters $\phi^{*}$ and $\theta^{*}$ as follows.

$$\phi^{*},\theta^{*}=\arg\min_{\phi ,\theta }-KL(q\|p)+E[\log p],$$ (11)

When those parameters are optimized, the negative KL divergence is minimized and the anticipated log-likelihood term is maximized.

#### 4.3.5 Loss function.

The main goal of this function, which is defined as *L*_*r*_ is to maximize the evidence lower bound (ELBO), which is equivalent to lowering the reconstruction loss and the KL divergence between the projected latent probability and the original distribution (the standard Gaussian).

$$L_{r}=-\log(p(x|z)).$$ (12)

The total loss for training the VAE is the sum of these two components. Overall, the working mechanism of our VAE model can effectively reduce the data dimensionality, ensuring the data is saved with the same input features by reconstructing the new data. This contribution leads to understanding complex relationships by visualizing and identifying new patterns and trends. Our VAE model can pinpoint the user-requested data and detect any kinds of false or erroneous requests by following the decoder reconstruction mechanism.

### 4.4 ML models

Following the application of the VAE for data representation, we later use different ML algorithms to execute the necessary goal of classifying the communication service for making the system an automated classifier.

The KNN algorithm categorizes data points based on their nearest neighbors [38]. For continuous data, Gaussian Naive Bayes makes use of Gaussian distribution assumptions [39]. Random Forest is a classification method that incorporates randomized decision trees whereas decision trees are adaptable for classification and regression [40,41]. Bagging classifier mainly approaches avoiding overfitting by averaging predictions from several models, whereas AdaBoost works by giving more weight to difficult-to-classify situations and less weight to those that are already handled well [42–44].

### 4.5 Neural network models

Following that, the complexity of the dataset is carefully considered in later utilizing neural network models. The method involves dividing the data into digestible portions, allowing for fast batch learning. The models include DNF network and MLP, both with the required layer architecture [45,46]. These models are trained for 30 epochs, with early stopping approaches used to improve their performance.

### 4.6 Proposed model

We present a lightweight CNN architecture, designed to streamline complex operations and services to the application, to improve the efficiency, speed, and reliability of our automatic network slicing allocation within the INBSI system.

This modification adds powerful feature extraction capabilities to the architecture, enabling rapid deployment and efficient application in classification tasks. To increase its performance, the proposed CNN model makes use of specified parameters. To capture extensive information patterns, the model begins with a convolutional layer with 64 filters and a kernel size of (3,1), and applies the ReLU activation function (ReLU). Using the same padding ensures that the dimensions remain consistent across the network. As a result, a max-pooling layer with a pool size of (2,1) dramatically lowers spatial dimensions. To boost the model’s representational capacity, we add a 128-filter convolutional layer with the same (3,1) kernel size and the same padding. This additional layer boosts feature extraction capabilities. Following these convolutional layers, we apply a flattened layer to the data to reshape it. This is followed by a thick layer with 256 neurons and ReLU activation. To prevent overfitting, a dropout layer with a rate of 0.5 is incorporated for regularization. Another dense layer with 128 neurons follows, adding another degree of feature abstraction. Finally, a dense layer with 64 neurons is added. The output layer implements the softmax activation function, simplifying multiclass classification jobs with three output classes. We equip the model with a more advanced and customized optimizer, “NADAM”, and a learning rate of 0.001.

To increase the performance of our model we use NADAM optimizer. The nesterov accelerated gradient (NAG) and Adam optimization procedures are combined in the NADAM optimization methodology. During training, the NADAM method is used to optimize the parameters of an ML model. It is particularly effective with neural networks and other deep-learning approaches. These are the steps that the algorithm takes.

Firstly, it calculates the gradient *g*_*t*_ of the loss function *f*_*t*_ with respect to the parameters $\theta_{t-1}$.

$$g_{t}=\nabla f_{t}(\theta_{t-1}),$$ (13)

Then by taking a weighted average of the preceding moment *m*_*t*−1_ and the gradient, you can update the first moment vector *m*_*t*_. This stage is analogous to how momentum operates in the NAG.

$$m_{t}=\beta_{1,t}m_{t-1}+(1-\beta_{1,t})g_{t},$$ (14)

Update the second-moment vector *n*_*t*_ by taking a weighted average of the previous second moment *n*_*t*−1_ and the square of the gradient $g_{t}^{2}$, where $\beta_{2}$ is the squared gradient smoothing parameter at time step *t*.

$$n_{t}=\beta_{2,t}n_{t-1}+(1-\beta_{2,t})g_{t}^{2},$$ (15)

Determine a temporary momentum correction (m) using the ratio of *m*_*t*_ to the sum of *i* for *i* ranging from 1 to *T* (where *T* is the number of time steps). This modification takes into account the prior instant values.

$$\hat{m}=\frac{\beta_{1,t+1}m_{t}}{1-\prod_{i=1}^{T}\beta_{1,i}}+\frac{(1-\beta_{1,t})g_{t}}{1-\prod_{i=1}^{T}\beta_{1,i}},$$ (16)

By generating a temporary squared gradient adjustment *n* based on the ratio of *n*_*t*_ to (1 - *n*) (where *t* is the squared gradient’s exponential decay).

$$\hat{n}=\frac{\beta_{2,t}n_{t}}{1-\beta_{2,t}},$$ (17)

Using the adjusted moment (*m*), squared gradient (*n*), and the learning rate (*t*), update the model’s parameters (*t*) where *t* is the learning rate at time step *t* and *ε* is a small constant to prevent division by zero.

$$\theta_{t}=\theta_{t-1}-\frac{\eta \cdot m_{t}}{\sqrt{n_{t}}+\epsilon },$$ (18)

**Fig 7:**
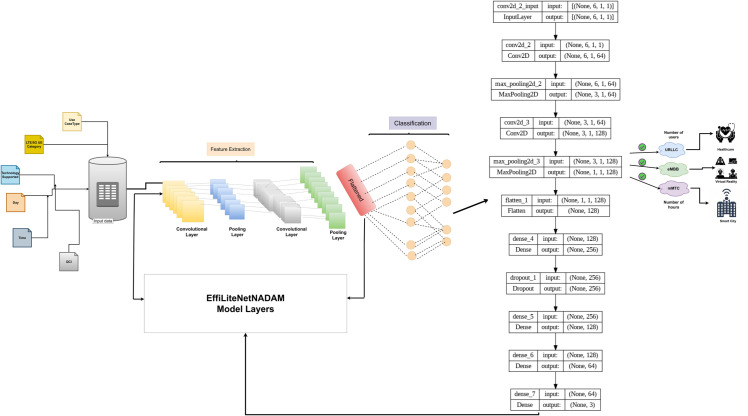
The proposed Model Architecture using NADAM Opitmizer.

The model architecture has been displayed in Fig 7. By implementing this proposed method with NADAM optimizer, the performance with classification will be much more efficient, and the end objective will be ensured by giving the correct slice type to the required applications. With this proposed DL method, the network operators can efficiently distribute slices toward applications and maintain load balancing for applications in an adaptive fashion. By receiving more data, the model will learn and make suitable decisions based on the determining input features. Our proposed model can capture spatial pieces of information based on the data provided process. If computation and prediction are required for a single slice, our suggested approach allows for flexibility, removing the requirement to train the complete dataset every time.

### 4.7 Interpretability methods used in network slicing

Even though our proposed classifier is designed to ensure the preservation of QoS criteria and efficient slice distribution inside our proposed INBSI process, the interpretability of the classification findings is imperative. As a result of the wide range of applications, each with its own set of requirements and technological settings, including communication protocols such as long-term evolution for machines (LTE-M) and narrowband Internet of Things (NB-IoT), this requirement arises.

#### 4.7.1 LIME.

In addition to being versatile, we utilize the LIME method for each slice to the black-box classifier, enabling users to construct explanations for specific predictions. To apply LIME in our study, we utilize XGBoost and our proposed CNN model as the underlying classifier due to its efficiency and strong performance in structured data classification. Therefore, it approximates the black-box model locally, despite its global limitations [47]. The general formula of LIME is as follows [48].

$$\Phi (x)=\alpha \cdot argmin\;\zeta (f,g,\pi_{x})+\Omega (g),$$ (19)

where *g* defines the presence of components that can be understood, Ω(g) signifies complication that contrasts with interpretability, $\pi_{x}(z)$ focuses locality between *z* to *x*, and *f*(*x*) defines the likelihood that *x* belongs to a class. Lastly, $\;\zeta (f,g,\pi_{x})$ illustrates how inaccurate *g* is in calculating *f* in the vicinity determined by $\pi_{x}$.

#### 4.7.2 SHAP.

For interpretation testing with addition global importance analysis, we apply SHAP to analyze feature importance in our classification model. To compute SHAP values, we again utilize the XGBoost and our proposed CNN model as our base classifier. XGBoost enhances interpretation through decision trees, leveraging unique tree learning techniques, logical measure sketching, and parallel computing for better scalability. It also integrates second-order derivatives to minimize model error effectively [50]. The Shapley value is the mean of the marginal contributions for all possible feature permutations [49]. The mathematical expression is as follows:

$$\emptyset_{i}=\sum_{S\subseteq N⧵\{i\}}\frac{|S|!(n-|S|-1)!}{n!}[v(S\cup \{i\})-v(S)],$$ (20)

where $\emptyset_{i}$ is the main focus of feature *i*, *N* is the set consisting of all the attributes, *n* is the number of features in *N*, *S* is the subset of *N* that contains feature *i*, and *v*(*N*) is principal base value, defining the predicted result for each attribute in *N* without knowledge of the feature values. The SHAP value of each feature for every analysis is included to estimate the model results for each observation. For a model *f* and feature vector *z*, the model is defined as follows.

$$g(z^{\prime })=\emptyset_{0}+\sum_{i=1}^{M}\emptyset_{i}z_{i}^{\prime }.$$ (21)

where *g* mainly refers to the explanation model, $z^{\prime }\in \{0,1{\}}^{M}$ primarily defines the feature vector of $z\text{ (so }z=h(z^{\prime })\text{)}$. *M* is the count of features and $\phi_{i}$ can be obtained from Eq 20. $\phi_{0}$ is the model output when all the

features are not present e.g., $z^{\prime }=h(0)$.

## 5 Performance evaluation metrics

Performance evaluation measures are used to assess the efficacy of each model. These include accuracy curves, confusion matrices, classification reports, MSE, along MAE.

As the accuracy curve indicates the model’s best possible accuracy, its smoothness represents its ability to classify. A smoother slope indicates a better classifier. We can easily track which models are correct and which are incorrect by comparing the actual labels to the projected labels in a confusion matrix.

The classification report for each model offers four metrics to evaluate its effectiveness: Support, Precision, F1 score, and Recall. Precision (P) is defined as the proportion of accurately anticipated outcomes of the total number of positively classified observations. In other words, it evaluates the accuracy of predictions and is formally stated as follows.

$$P=\frac{T_{p}}{T_{p}+F_{p}},$$ (22)

The recall (R) metric is determined by dividing the total number of initial class evaluations by the number of anticipated results.

$$R=\frac{T_{p}}{T_{p}+F_{n}},$$ (23)

The *F1*-score is calculated by averaging precision and recall.

$$F1=\frac{2\times precision\times recall}{precision+recall},$$ (24)

where *T*_*p*_ is True Positive, *F*_*p*_ is False Positive, *F*_*n*_ is False Negative, and *T*_*n*_ is True Negative.

MSE quantifies the level of error in statistical models. It computes the average squared difference between observed and predicted data. When a model has no mistakes, the MSE is zero. As model inaccuracy grows, so does the value.

$$\text{ MSE }=\frac{1}{n}\sum_{i=1}^{n}{\left(y_{i}-{\hat{y}}_{i}\right)}^{2},$$ (25)

MAE measures the error difference between two observations of the same event, used in comparisons like predicted versus observed, subsequent time versus starting time, and different measuring techniques.

$$\text{MAE}=\frac{1}{n}\sum_{i=1}^{n}\left|y_{i}-x_{i}\right|,$$ (26)

## 6 Result and analysis

### 6.1 Hyperparameters and reconstruction error calculation of VAE

A validation dataset is used to assess the VAE’s performance, and a baseline loss is calculated. Reconstruction skills and data representation effectiveness are evaluated using model loss, mean squared error, and absolute error. The metrics are calculated and given in Table 3. Visualization and anomaly detection indicate VAE’s performance, with lower MSE values indicating better data reconstruction and average MAE values indicating accurate predictions.

**Table 3:** VAE performance baseline.

Evaluation Metric	Result
Baseline Loss (MSE)	0.083
Model Loss (MSE)	0.917
Model MAE	0.006

Table notes: MSE = Mean Squared Error, MAE = Mean Absolute Error. The VAE shows higher model loss due to reconstruction differences but maintains low absolute error.

Fig 9(a), 9(b) mainly depicts the loss curve and a comparison of original and rebuilt data. This decrease or stabilization of the loss curve suggests that the VAE model has learned to encode the input data into a latent space representation and is successfully reconstructing the original input from this latent representation. The reduction in loss in Fig 9(a), 9(b) suggests that the model is slowly closing the gap between the reconstructed samples and the original input data.

**Fig 8:**
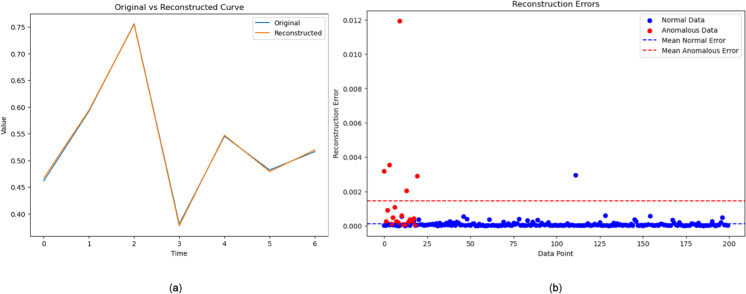
Anomaly detection of the constructed data.

Fig 8(a), 8(b) depicts the similarities between the reconstructed and original data. Our implemented VAE model accurately recovers original data from learned representations, demonstrating its ability to capture and recreate underlying patterns and structure in data. This discovery has implications for occupations like data generation and anomaly detection in network slicing.

**Fig 9:**
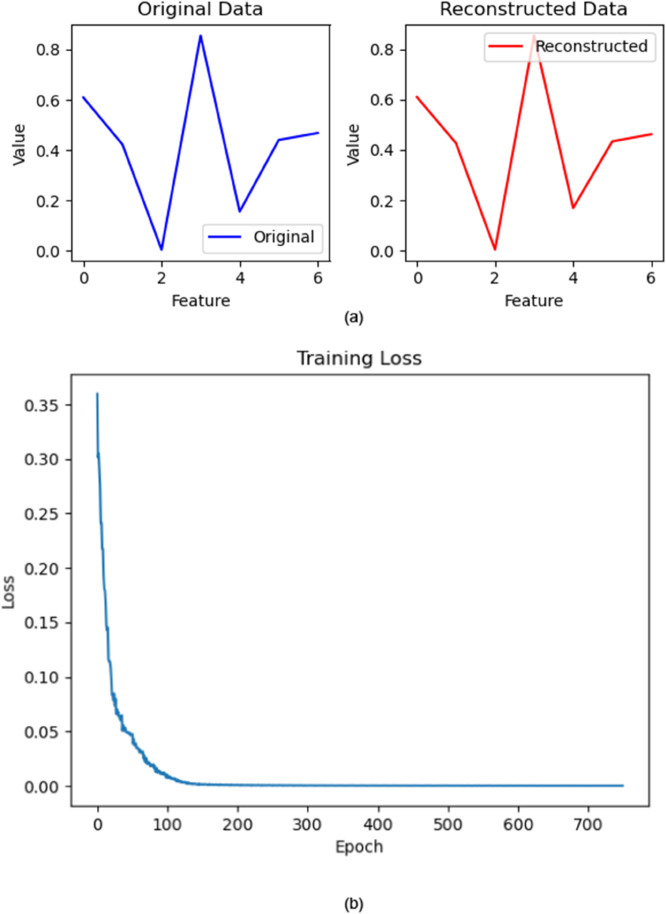
Training Loss of VAE.

### 6.2 ML results

**Fig 10:**
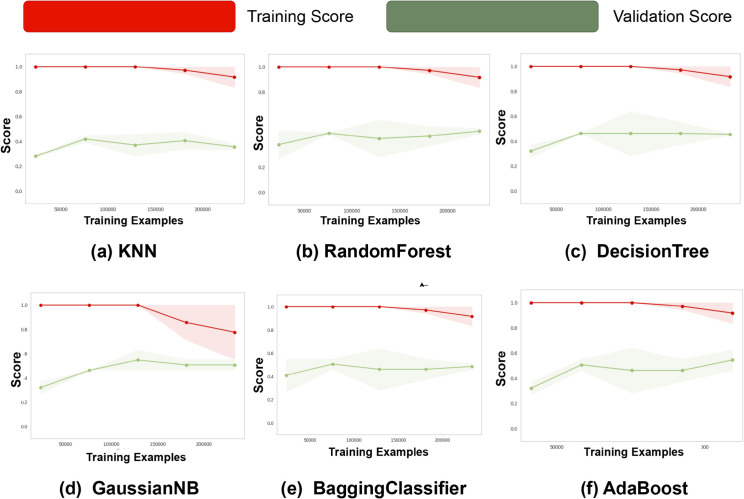
Learning curves for the ML models. Learning curves demonstrate model stability with increasing data, critical for dynamic slicing where traffic patterns evolve for machine learning models.

Fig 10 illustrates the learning curves of six classifiers, showing their generalization behavior. Random Forest and Bagging Classifier demonstrate strong performance with converging training and validation scores, indicating good generalization. KNN and Decision Tree exhibit overfitting, with high training accuracy but poor validation performance. Gaussian Naive Bayes shows underfitting, as both scores remain low and close. AdaBoost improves with more data, gradually narrowing the gap between training and validation scores. Overall, ensemble methods like Random Forest and Bagging are more robust, while simpler or unregularized models tend to overfit or underfit.

**Fig 11:**
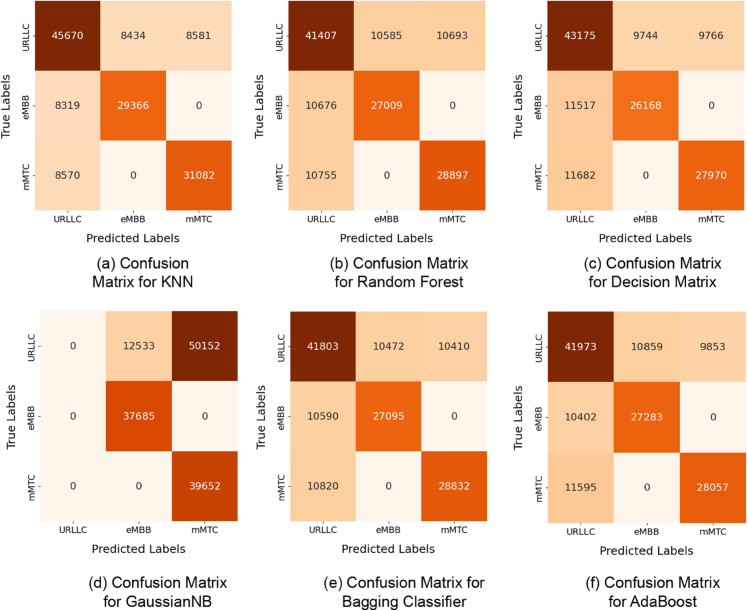
Confusion Matrices for the ML models.

The confusion matrix is shown in Fig 11, and it illustrates how well the model performed for each class. The confusion matrix shows that the models give consistent results across classes, demonstrating consistent performance over a wide range of classifications. These matrices can be examined to discover intriguing patterns and misclassifications within specific classes. Except for the GaussianNB model is presented in Fig 11(d), all of the models perform well for the target feature, although with some misclassifications. As illustrated in Fig 11(b), 11(c), 11(e), 11(f), there are additionally some misidentifications in Random Forest, Decision Tree, Bagging Classifier, and AdaBoost.For the model, the “eMBB” communication service was misclassified as URLLC, with a 40–45 instance range.

**Fig 12:**
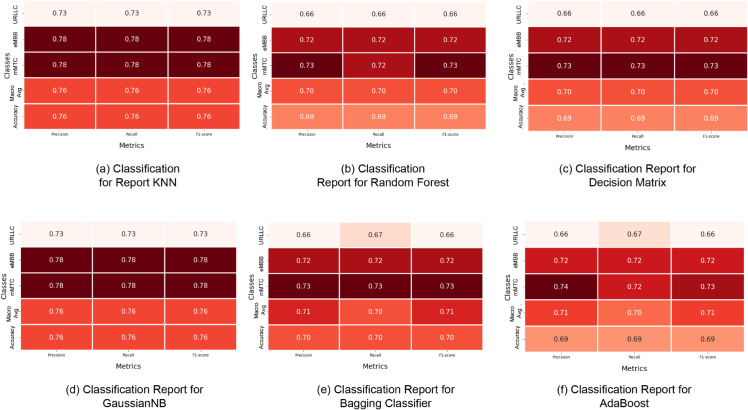
Classification Report for the ML models.

Fig 12 also shows the classification report for each model in addition to the learning curve. All the classifier’s results are being shown. In between, from Fig 12(b), 12(c), 12(e), 12(f), the random forest, decision tree, bagging classifier, and AdaBoost relatively show less performance than the other two models shown in Fig 12(a), 12(d). The precision, recall, and f1-score for the KNN of mMTC class is 78%, which is much higher than the other adjacent models. The performance metrics for the decision tree and AdaBoost are relatively lower with the values of 66%−67% range of precision, recall, and F1-score for the URLLC class.

### 6.3 DL results

**Fig 13:**
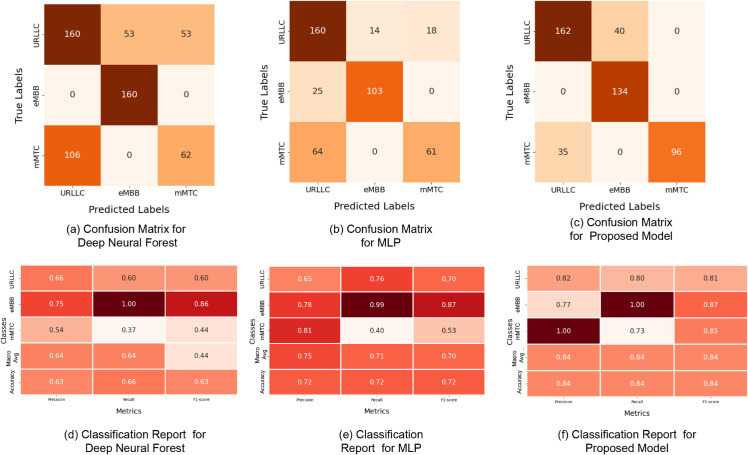
Confusion Matrix and Classification report for DL models and Proposed CNN Model.

The confusion matrix of the proposed model provides useful information on how the DNF model, MLP model, and recently introduced model perform. According to the research, there are misclassifications, particularly the predicted communication services in Fig 13. Several misclassifications are found in the DNF model, including the incorrect categorization of “mMTC” and “URLLC” services. Despite these errors in classification, the suggested model outperformed others. There are significantly fewer misclassifications of communication services due to the proposed model’s accuracy in identifying and differentiating them. Our proposed model properly identifies each communication service as one of the sliced target labels.

For each model, Fig 13 displays the classification report by class together with the relevant confusion matrices discovered during model training. According to the DNF model, the precision and f1-score of the mMTC class are much lower than those of the other two classes. Accordingly, the DNF model has difficulty correctly categorizing cases that fall under the “mMTC” category. The “URLLC” class also performs poorly in the DNF model, suggesting it may be difficult to distinguish “URLLC” instances from others. To classify “mMTC” and “URLLC” communication services appropriately, the DNF model needs to be further developed. A similar set of performance issues can also be seen in the MLP model for the “mMTC” class. As compared to other classes in the MLP model, the “mMTC” class shows recall and f1-score are noticeably lower. Therefore, the MLP model may have difficulty identifying cases that fall under the “mMTC” category. However, the proposed model outperforms the previous models in terms of precision, recall, and f1 scores, indicating that improvements are needed to properly categorize “mMTC” communication services. According to the proposed model, the “mMTC” class has a higher f1 score than the other classes.

**Fig 14:**
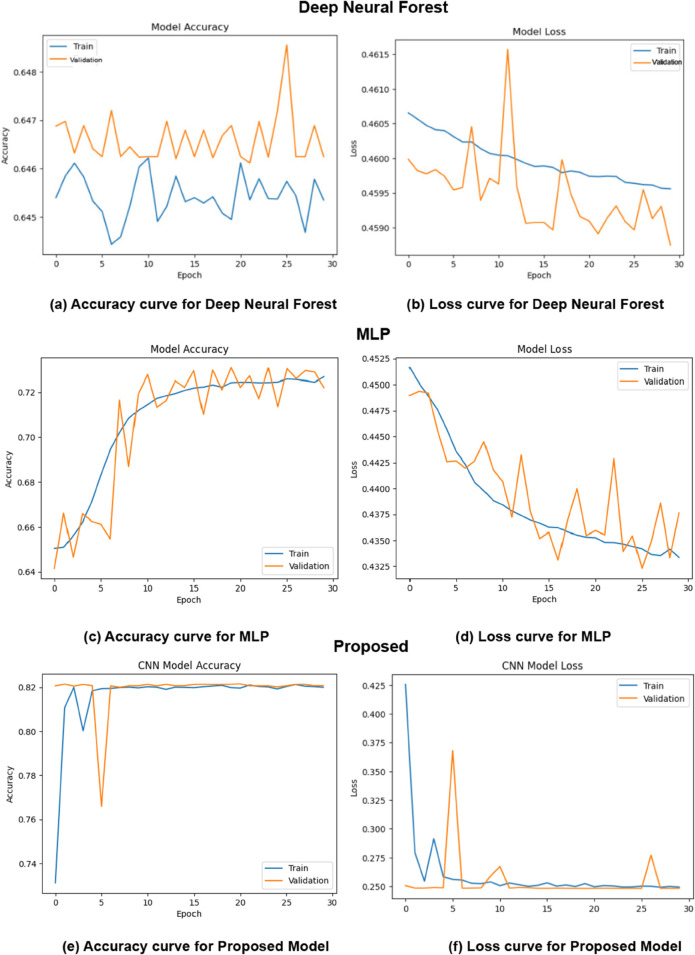
Training accuracy and loss curves for the DL models with our proposed model.

The training progress over 30 epochs with early stopping is shown in Fig 14, along with the accuracy and loss curves for the chosen models. Through the analysis of these curves, we can better understand the DNF model, the MLP model, and our proposed model. The DNF model’s training accuracy curve reaches 64%, but oscillations indicate instability due to local maxima interactions and limited generalization abilities with training and validation data.

In contrast, the training and validation accuracy curves of the MLP model show fair results after obtaining a training accuracy of 65%, the graphs indicate a positive increasing trend, indicating that the model is steadily improving its results on both the training and validation data than other models. Our proposed model shows promising results based on the accuracy curves. Training and validation curves show positive trends, indicating consistency in accuracy gains over time.

### 6.4 Performance comparison of algorithms and models

**Fig 15:**
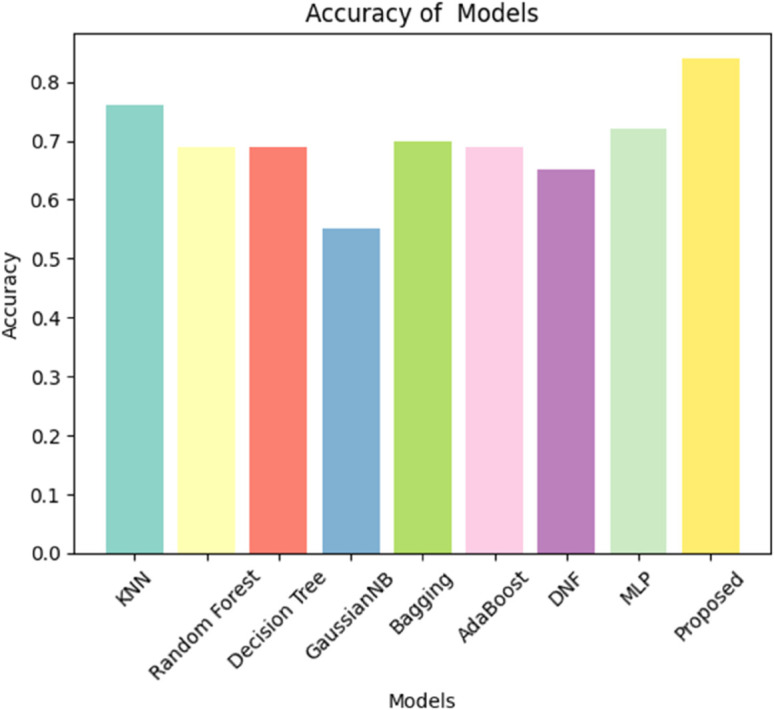
Comparison of the models.

**Fig 16:**
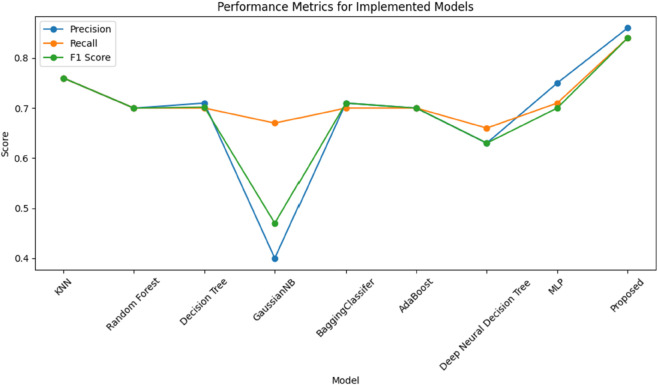
Achieved scores of these models based on performance metrics.

**Fig 17:**
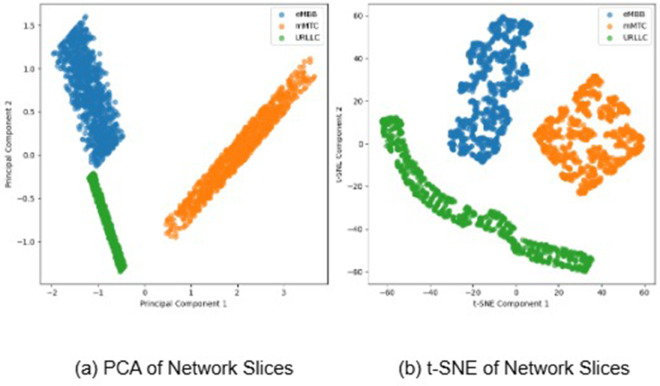
2D PCA and t-SNE plots showing distinct clusters for network slices, indicating effective feature extraction and slice differentiation by the INBSI model.

**Fig 18:**
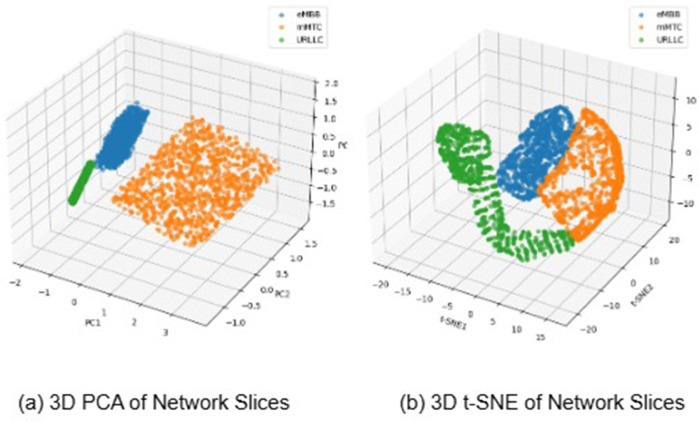
3D PCA and t-SNE plots demonstrating well-separated clusters for network slices, further validating the INBSI model’s ability to learn meaningful, discriminative feature representations.

In our comparison research, we apply a set of models to test the effectiveness of our proposed model in identifying three separate communication services: eMBB, URLLC, and mMTC. The models under examination include KNN, Random Forest, Decision Tree, GaussianNB, Bagging, AdaBoost, Decision Forest Tree, and MLP. These models are selected based on their shown efficacy in a range of categorization tasks, as illustrated in Fig 15. In addition, Fig 16 shows a plot with models on the x-axis and scores on the y-axis. The plot’s three lines show the precision, recall, and F1 scores for each model. Our proposed model appears to be most effective in all three criteria (precision, recall, and F1). The Random Forest, AdaBoost, and BaggingClassifier models also perform well, with scores nearly identical to one another and slightly over 0.7 for the majority of standards. The GaussianNB and DNF appear to perform much less than the others. To improve accuracy and use the distinct capabilities of independent models, our proposed model is intended for robust feature extraction and classification applications in the INBSI system. We further visualize the feature distributions of our INBSI model using 2D and 3D principal component analysis (PCA) and t-distributed stochastic neighbor embedding (t-SNE) plots. In the 2D and 3D PCA plots, network slices form distinct clusters, showing that the model effectively captures key features like bandwidth, latency, and packet loss. Minimal overlap indicates strong slice differentiation shown in Figs 17 and 18. The 2D and 3D t-SNE plots further confirm this, with well-separated clusters demonstrating the model’s ability to learn meaningful representations. The clear separability highlights the effectiveness of our CNN-based feature extraction and interpretability tools. Therefore, our proposed model adapts well to unexpected validation data and learns effectively from the training data. On both the training and validation datasets.

Experiment results, which are shown in Table 4, illustrate that these models are effective at solving the stated function. KNN achieved a 76% accuracy rate, demonstrating its capacity to discover patterns in datasets. Both the Random Forest and Decision Tree models achieved 69% accuracy, demonstrating their ability to deal with difficult decision boundaries. GaussianNB obtained 55% accuracy, demonstrating the challenge’s limitations.

**Table 4:** Comparison of the models.

Model	Acc(%)	Precision	Recall	F-1
**Traditional ML Models**				
KNN	76	0.76	0.76	0.76
Random Forest	69	0.70	0.70	0.70
Decision Tree	69	0.71	0.70	0.702
GaussianNB	55	0.40	0.67	0.47
BaggingClassifier	70	0.71	0.70	0.71
AdaBoost	69	0.70	0.70	0.70
**Neural Network Models**				
Deep Neural Forest	65	0.63	0.66	0.63
MLP	72	0.75	0.71	0.70
**Proposed Model**				
NADAM Optimized CNN	**84**	**0.86**	**0.84**	**0.84**

The ensemble-based learning accuracy of Bagging Classifier and AdaBoost is high, while the DNF model obtains 65% accuracy. The MLP model captures complex information relationships with 72% accuracy.

Table 5 compares the inference times of two neural network models, highlighting the efficiency of the proposed approach. The NADAM-optimized CNN achieves the lowest inference time of 3.41 milliseconds, significantly outperforming the standard MLP, which requires 5.82 milliseconds. This improvement underscores the effectiveness of using the Nesterov-accelerated Adaptive Moment Estimation (NADAM) optimizer in enhancing model efficiency, making the proposed CNN architecture more suitable for real-time or resource-constrained applications. Besides, the proposed NADAM Optimized CNN model demonstrates statistically significant improvement over MLP and DNN, with the lowest p-value (0.012) and highest chi-square score (6.89). These results confirm the superior reliability and performance consistency of the proposed architecture in Table 6.

**Table 5:** Inference Time Comparison of Models (Lower is Better).

Model	Inference Time (ms)
**Neural Network Models**	
MLP	5.82
**NADAM Optimized CNN (Proposed)**	**3.41**

**Table 6:** Statistical Analysis of Model Performance.

Model	p-value	Chi-square ($\chi^{2}$)
**Neural Network Models**		
MLP	0.037	4.32
DNN	0.024	5.16
**NADAM Optimized CNN (Proposed)**	**0.012**	**6.89**

### 6.5 Interpreted results and insights

#### 6.5.1 LIME.

**Fig 19:**
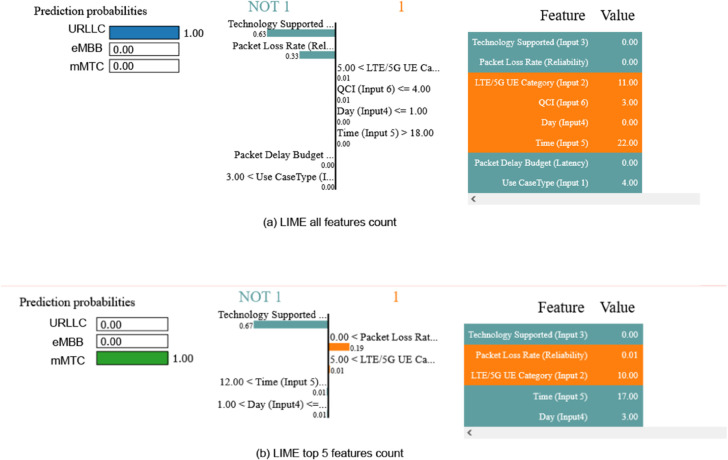
LIME interpretability with decision tree.

**Fig 20:**
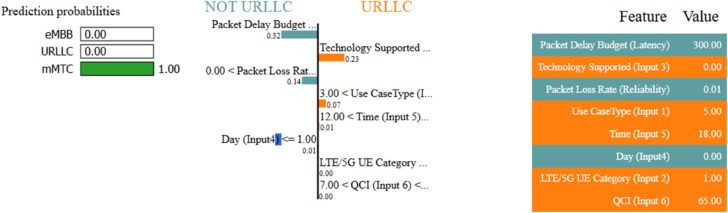
LIME interpretability with our proposed CNN.

In this section, we look at the LIME explanations. Figs 19 and 20 depict these explanations in three sections: prediction probabilities for all possible outputs, bar charts illustrating feature weights and contributions, and a feature value table. The “1” labeled side mainly focuses on the correct prediction based on the input feature’s importance, and “NOT 1” does vice versa. The decision tree classifier properly predicted the outcome for a specific input data point, as shown in Fig 19, and the MLP classifier for Fig 22.

The prediction probability for the target feature “eMBB” is 1.00, indicating high confidence in this prediction. The odds for the other two target qualities, on the other hand, are 0.00, meaning that they will not be expected outcomes. This gives an accurate prediction allocation for the network infrastructure. The feature value table reveals the Decision Tree classifier’s accuracy, with blue values supporting “URLLC” and white values supporting the opposite prediction. Blue marks indicate the target slice, while white colors indicate other slicing. Features “eMBB” and “mMTC” accurately identify outputs. The predicted output “URLLC” is accurately classified by the “LTE/5G UE Category” and “Time” feature values. However, the parameters “Technology Supported” and “Packet Loss Rate” negatively impact the prediction, suggesting alternative communication services like eMBB or mMTC. Using the top five attributes in Fig 20(b), we investigate the projected output deeper. As a result, the characteristic “2” as “mMTC” which symbolizes “mMTC” is correctly discovered. “Packet Loss Rate (Reliability)” and “LTE/5G UE Category (Input 2)” as The Long-Term Evolution(LTE) are impact factor features, with “LTE/5G UE Category (Input 2)” having a value of 10.00. In contrast, the feature “Technology Supported (Input 3)” has a negative impact factor with a value less extensive than the threshold, precisely –0.67.

**Fig 21:**
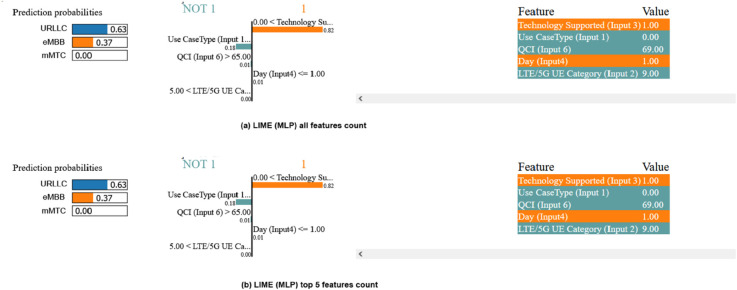
LIME interpretibility with MLP.

We additionally employ the LIME with MLP method, as shown in Fig 21. The LIME with MLP approach is used in the study to evaluate class label predictions using input features, with a focus on “Technology Supported” measures for CNN models to ensure accurate slice distribution by knowing the correct type configuration for individual users in the system.

The LIME explanation reveals that our proposed model 21 predicts mMTC (1.00 probability) with high confidence, driven primarily by the Packet Delay Budget (latency = 300.00, contribution: 0.32) and Technology Supported (contribution: 0.23), while Packet Loss Rate (0.01 value, contribution: 0.14) also plays a supporting role. Features like Use Case Type (5.00 value) and QCI (65.00) align with mMTC’s profile (e.g., tolerant to latency but sensitive to interference). In contrast, negligible contributions from Time (0.01), Day (0.01), and UE Category (0.00) confirm they had little impact on the prediction. This breakdown validates the model’s focus on latency and reliability metrics for mMTC classification, consistent with typical IoT network behavior.

#### 6.5.2 SHAP.

**Fig 22:**
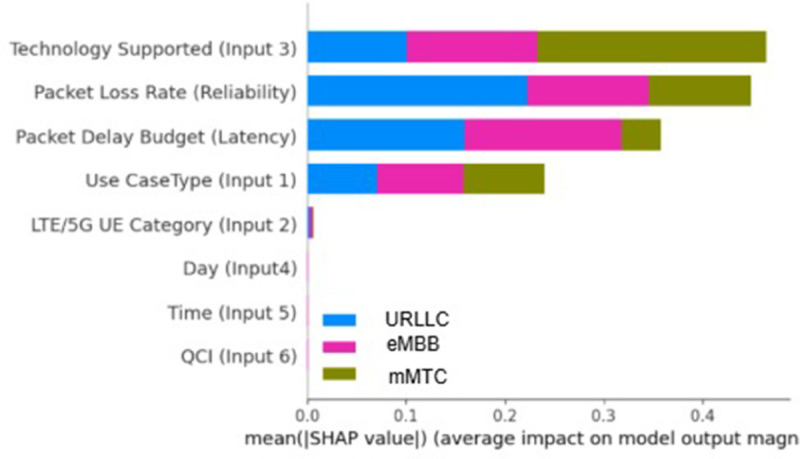
SHAP feature importance and summary plot of proposed CNN.

**Fig 23:**
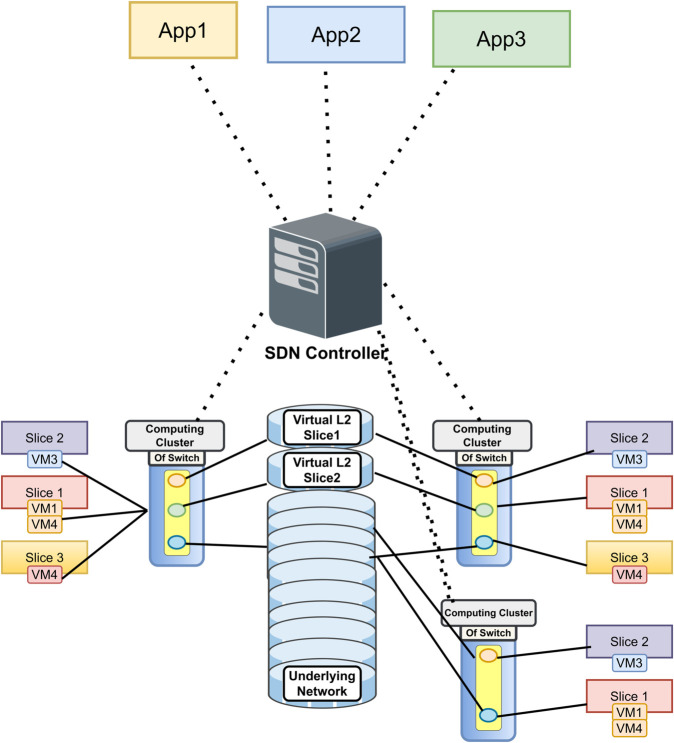
SHAP feature importance and summary plot of proposed CNN model.

SHAP is used to infer the model’s predictions. In addition to making use of Shapley values, the SHAP library provides a powerful tool for examining how different variables affect the model’s predictions. SHAP ranks features based on average SHAP values, highlighting those that are crucial for model prediction. Fig 22(b) shows a more detailed breakdown of how each attribute affects certain outcome labels. On the Y-axis, features are listed according to their average absolute SHAP values. The values on the X-axis represent SHAP values that have the greatest impact on model output. From one correlation to another, the predicted value on the specific feature is derived from the central zero value. Positive values for a particular characteristic move the prediction of the model closer to the label being considered, which can be described as highly correlated. In Fig 23, the “Technology Supported” and “Packet Loss Rate” features indicate a higher likelihood of influencing slice communication distribution. Based on these communication protocols, the model can precisely distribute the appropriate slice-type service to the specific application.

**Fig 24:**
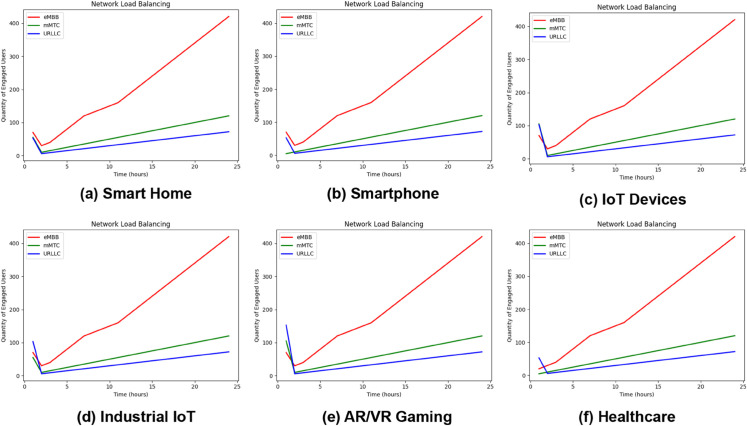
The tree-structured output of the XGBoost classifier on the SHAP model.

Besides, in Fig 24, the decision-making process of our SHAP model, where we use XGBoost as a base classifier, is represented as a tree structure, where each node corresponds to a feature split. For instance, the model first evaluates the “Packet Loss Rate (Reliability)” feature, determining whether it exceeds a specific threshold. Based on this decision, the process proceeds to the appropriate branch until a final classification is reached. By applying SHAP to this model, we can quantify the contribution of each feature to a given prediction, providing insights into the classifier’s decision-making process.

**Fig 25:**
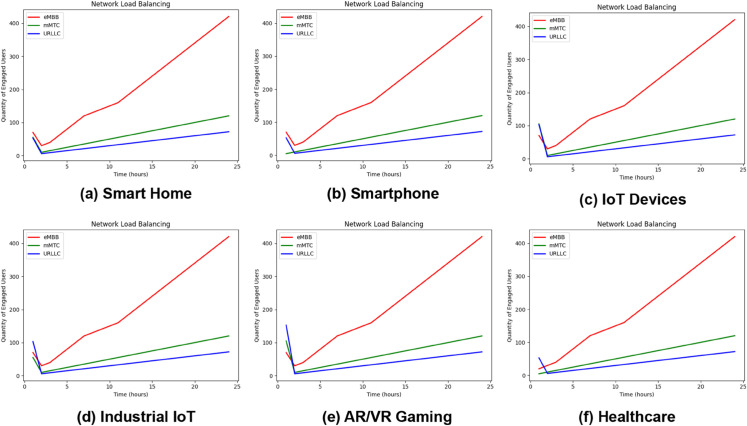
Slice distribution using our proposed hybrid CNN model.

Finally, we evaluate our model with the proposed approach, which is shown in Fig 25. The SHAP analysis reveals that Use Case Type (Input 1), Packet Loss Rate (Reliability), and Packet Delay Budget (Latency) are the most influential features in the model, with the highest mean absolute SHAP values, indicating their strong impact on predictions. Features like Technology Supported (Input 3) and LTE/5G UE Category (Input 2) contribute moderately, while Day (Input 4), Time (Input 5), and OCI (Input 6) have relatively minor effects. This suggests that network performance predictions are primarily driven by application requirements (URLLC/eMBB/mMTC) and quality-of-service metrics (loss rate, delay), while temporal and interference factors play lesser roles. The results align with expected network behavior, validating the model’s interpretability.

**Algorithm 1. Advanced slicing distribution process in INBSI pipeline.**

1: Initialize the proposed model $M_{\text{proposed}}$

2: Initialize slice services $S_{\text{service}}$

1: User demand service $R_{\text{user}}(p_{1},\ldots ,p_{n})$ with requirement parameters

4: **for** *i* = 1 to $N_{\text{requests}}$ **do**

5:   Initialize VAE model $V_{\text{VAE}}^{\left(i\right)}$

6:   **for** *j* = 1 to $N_{\text{requests}}$ **do**

7:    $U_{\text{data}}.\text{validate}(V_{\text{VAE}}^{\left(i\right)})$

8:    **for** each user $u\in U_{\text{data}}$

9:     **if** user has special requirements **then**

10:      $u_{\text{model}}\leftarrow \text{customize}(U_{\text{data}})$

11:     **else**

12:      $u_{\text{model}}\leftarrow \text{train}(U_{\text{data}})$

13:     **end if**

14:     ${\hat{y}}_{\text{slice}}\leftarrow \text{predict}(S_{\text{type}})$

15:     $U_{\text{data}}\leftarrow \text{interpret}({\hat{y}}_{\text{slice}})$

16:    **end for**

17:   **end for**

18:   Users send updates to applications

19:   **if** additional requests pending $N_{\text{pending}}>0$ **then**

20:    handleAdditionalRequests()

21:   **end if**

22:   **if** system load $L_{\text{system}}>T_{\text{load}}$ **then**

23:    adjustResourceAllocation()

24:   **end if**

25: **end for**

## 7 Use scenarios and performance assessment of the proposed model

When led by predictions, load balancing can optimize resource allocation, reduce congestion, and improve user experiences. Based on the scenario, Fig 25 primarily depicts our trained proposed model used to simulate load balancing dynamically across various network service types using the given input features. We take some applications as defined in the “Use CaseType” input feature. For each application, we simulate the results by updating the load for each slice type. For example, Smart Home, Smartphone, IoT Devices, Industry IoT, AR/VR Gaming, and Healthcare. Fig 25(a) shows the load balancing scenario for Smart Home. Based on the number of users, the application changes its service type over time, and this is happening due to the contribution of our proposed model. Same cases for other scenarios in Fig 25(b), 25(c), 25(d), 25(e), and 25(f). It illustrates the outcomes of the load-balancing simulation. Over 24 hours, the output shows the load values as the number of active users for each service type (eMBB, mMTC, and URLLC). The variations in load levels anticipated by the model reflect the probable benefits of load balancing. Notably, at peak hours, the count of load numbers for eMBB and mMTC climb substantially, demonstrating effective load distribution. The proposed model efficiently controls workload distribution among application slices in the INBSI system environment, taking into consideration both demand and time limits. This system constantly reviews service performance by dynamically changing slices depending on varying load conditions, efficiently optimizing resource allocation across high-load and low-load scenarios.

We provide the “Advanced Slicing Distribution Process” in Algorithm 1 for the INBSI system pipeline, which outlines the procedures for initializing the proposed model, initializing slice services, and handling user demand services with requirement parameters. This approach iteratively initializes a VAE, examines user data, customizes models if necessary, predicts slice types, and interprets user input for a set number of requests. Additionally, it handles additional requests and modifies resource allocation based on system load.

We further compare our approach, specifically with VAE, for anomaly detection and interpretation testing using LIME and SHAP, as shown in Table 7. The existing approaches by Syed et al., Ramraj et al., Neha et al., and Sowmya et al. do not incorporate either VAE or XAI techniques, limiting their ability to enhance data validity and interpret model decisions. In contrast, our proposed approach integrates both VAE and XAI, ensuring improved data reconstruction, anomaly detection, and interpretability, which collectively contribute to a more robust and transparent network slicing classification system.

**Table 7:** Model comparison used.

Model	VAE Used	XAI Used
Syed et al. [32]	✗	✗
Ramraj et al. [33]	✗	✗
Neha et al. [34]	✗	✗
Sowmya et al. [35]	✗	✗
**Our Proposed Approach**	✓	✓

### 7.1 Limitations

While this study addresses class imbalance using SMOTE and ensures data privacy through anonymization, future research should explore dynamic imbalance mitigation techniques that adapt to the evolving traffic patterns in 5G and emerging 6G environments—particularly through adaptive resampling methods sensitive to temporal slice demand fluctuations. Ethical robustness can be further strengthened by employing federated learning to decentralize the processing of sensitive data, coupled with differential privacy techniques to safeguard slice-level analytics, especially as global regulatory frameworks like the EU AI Act continue to evolve. In terms of computational efficiency, incorporating quantum-inspired optimization [51] strategies could significantly enhance the scalability and responsiveness of network slicing mechanisms, offering a promising avenue for addressing complex resource allocation challenges. Furthermore, analyzing the energy-efficiency trade-offs of lightweight AI models in edge environments and ensuring their compatibility with zero-trust architectures defined by standardization bodies such as 3GPP would promote both sustainability and security. Finally, grounding these advancements within the broader context of future network visions underscores the necessity of designing AI systems that are not only technically capable but also aligned with long-term industry trajectories and societal expectations.

## 8 Conclusion and future work

Network slicing is considered to play an important role in future generations of communication networks. Modern wireless networks’ communication mechanisms are shaped by mathematical models that frequently lack accuracy. To extract features and classify network slices, we used our lightweight, tailored, optimized CNN model, making it suited for efficient slice distribution while ensuring load balancing in our INBSI system. With VAE, network slicing service efficiency could be evaluated along with fault detection and recovery, and LIME and SHAP techniques helped shed light on the decision-making process and highlighted specific input requirements. We contributed to the development of network-slicing technologies and guided network operators to improve their utilization plans through the results of our research. The proposed method improves network slicing through VAEs for anomaly detection, CNN with NADAM optimization, and interpretable AI approaches; yet, numerous problems persist. The computational complexity of VAEs may impede real-time implementation, necessitating model compression or hardware acceleration. Scalability is a critical issue, as effective resource allocation is essential for extensive 5G networks. Security continues to be a significant challenge, especially in safeguarding sensitive information. Federated learning can reduce privacy issues; nevertheless, it also presents vulnerabilities to model poisoning attacks. Robust encryption techniques, including homomorphic encryption, are essential for improving data security while maintaining efficiency. Future endeavors will concentrate on enhancing efficiency, scalability, encryption-based security, and resilient federated learning.
